# Synthesis of Magnetic Hyperbranched Star Chain Nanopolymer and Its Application in ASP Flooding Wastewater Treatment

**DOI:** 10.3390/molecules31111816

**Published:** 2026-05-25

**Authors:** Sanyuan Qiao, Luoqi Cui, Li Cai, Zhenzhong Fan

**Affiliations:** 1 Qinhuangdao Campus, Northeast Petroleum University, Qinhuangdao 066004, China; mrtatt@nepu.edu.cn; 2 School of Petroleum Engineering, Northeast Petroleum University, Daqing 163318, China; caili9875@163.com (L.C.); fanzhenzhong@163.com (Z.F.)

**Keywords:** magnetic hyperbranched molecule, cationic polymer, demulsification and flocculation, asp flooding sewage, sewage treatment mechanism

## Abstract

ASP flooding wastewater contains crude oil, suspended solids, anionic polymers and surfactants, with high viscosity, high zeta potential, difficult demulsification, flocculation and slow separation and sedimentation. In order to solve the problem of wastewater treatment of ASP flooding in oil fields, a magnetic branched core was prepared from ethyl silicate (TEOS), nano Fe_3_O_4_ and aminopropyl triethoxysilane (APTES), and then reacted with polyamine and methyl acrylate to synthesize the magnetic hyperbranched molecule FSNMN with demulsification ability. Using acrylamide (AM), acryloxyethyl trimethylammonium chloride (DAC) and maleic anhydride (MA) as raw materials, cationic polymer long chain (CAMHA) with flocculating properties was synthesized and grafted with hyperbranched molecules. The demulsification flocculation ability of the product regarding ASP flooding wastewater was evaluated, and the demulsification flocculation mechanism was summarized. The results showed that the average molecular weight of 3-FSNMN_4_-C was 4.7 million, the cationic degree was 20.5%, and the saturation magnetization was 20 EMU/g. The removal rate of oil and suspended solids was 93.82% and 91.95% respectively when the simulated sewage was treated by magnetic field for 30 min. Magnetic hyperbranched star chain polymer provides a solution to the serious ecological environment problems caused by ASP flooding.

## 1. Introduction

ASP flooding technology has been widely promoted and applied in many oilfields. Even under the extreme production condition of 98% water cut in the oil layer, ASP flooding technology can improve oil recovery by more than 20 percentage points [1]. In China alone, the application scope of ASP flooding technology has covered more than 200 million tons of geological reserves and contributed more than 10% of the annual production, but it also brought 100 million tons of ASP flooding sewage.

Oilfield sewage will cause harm to the function of oilfield reuse system [2]. As a valuable resource, the shortage of water resources is a worldwide situation. Selecting reservoir reinjection technology can effectively reduce the total water consumption in water shortage areas [3,4]. However, without effective treatment before reinjection, the internal emulsified oil, polymers and suspended solids may cause reservoir plugging, reservoir permeability and the recovery factor to decline [5]. The targets of ASP flooding wastewater treatment are soluble organic matter, oil phase, solid impurities and salt containing mixed phase. In order to remove these pollutants, conventional methods include flocculation and coagulation, sedimentation filtration, oxidation and electrochemical deposition, membrane treatment, etc. [6,7,8].

Hyperbranched polymer can be used as the main reagent to participate in the processes of filtration and precipitation, catalytic oxidation, adsorption and separation [9,10,11,12]. For ASP flooding wastewater treatment, more types of functional groups are required to participate in the structure of the treatment agent, and the treatment agent material needs to have a stable carrier structure [13,14]. The magnetic nano core can be used as the central core [15] and is rarely used in the field of ASP flooding sewage in the oilfield. This study explores its application in the field of ASP flooding sewage in the oilfield. Hyperbranched polymers (HBPs) have a high application potential due to their unique physical and chemical properties brought about by their highly branched structure [16,17,18].

For ASP wastewater with high viscosity and high stability [19], the magnetic sedimentation rate is far less affected by viscosity than the gravity sedimentation rate, and can achieve efficient separation within 30 min. However, the application of magnetic nanomaterials in ASP wastewater treatment is still very limited [20]; especially multifunctional materials combining a magnetic core with hyperbranched demulsifier and cationic flocculant have not been reported [21,22]. The purpose of this study is to synthesize a magnetic hyperbranched star chain nanopolymer with demulsification, flocculation and magnetic separation functions, which provides a new solution for the efficient treatment of ASP wastewater.

## 2. Materials and Methods

### 2.1. Materials

Tetraethyl silicate, ammonia, nano Fe_3_O_4_ (50 nm), methyl acrylate, ethylenediamine, 1,3-propanediamine, diethylenetriamine, triethylenetetramine, tetraethylenepentamine, 3-aminopropyltriethoxysilane, acrylamide, acryloyloxyethyl trimethylammonium chloride, maleic anhydride, and ammonium persulfate of analytical grade purity were the reagents purchased from Aladdin reagent (Shanghai) Co., Ltd. (Shanghai, China). Twain, Span, SDBS, and SAS of industrial grade purity were the reagents purchased from Shandong Yousuo Chemical Technology Co., Ltd. (Linyi, China). HPAM, non-PAM, oilfield bentonite of industrial grade purity, and oilfield supplies were purchased from ordinary sales platforms in China. All performance testing experiments were independently repeated 3 times.

### 2.2. Synthesis of Magnetic Hyperbranched Molecular Intermediates

**Figure 1:**
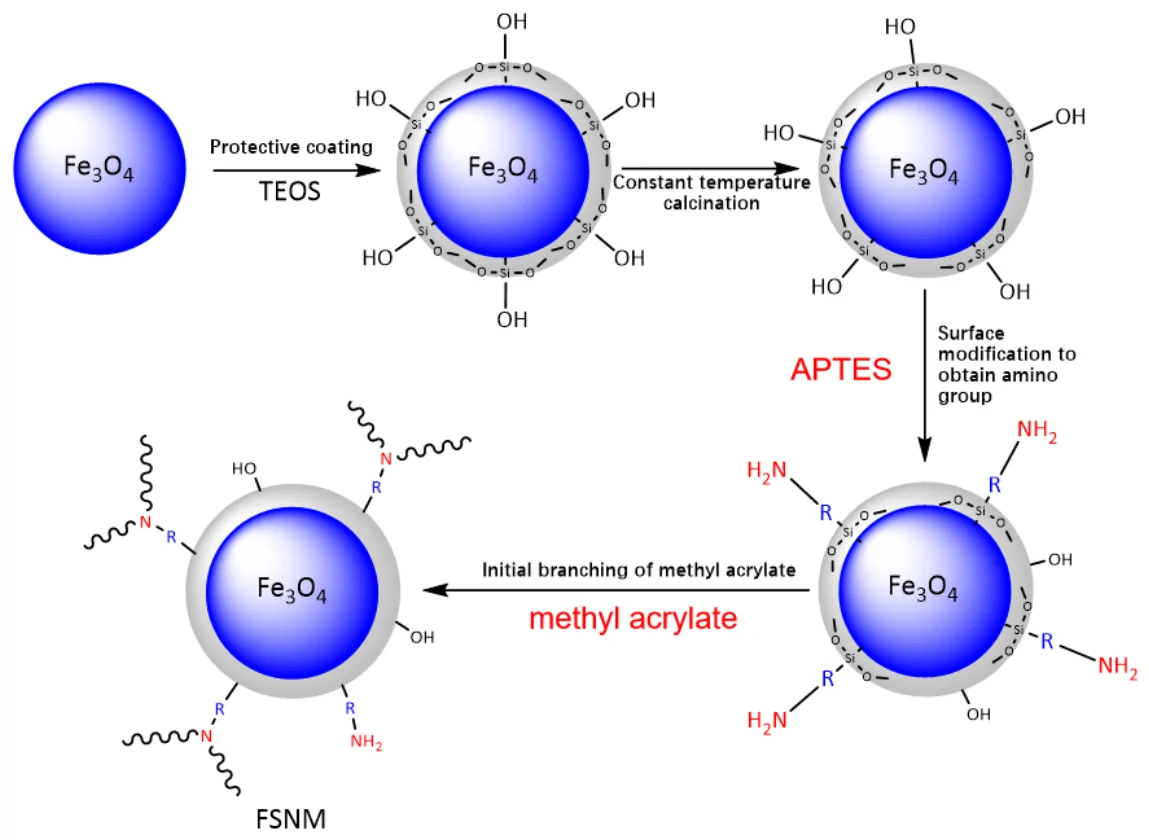
Synthesis route of FSNM.

The magnetic core material coated with SiO_2_ needs constant temperature vacuum drying treatment, and the basic material of the magnetic core is selected as Fe_3_O_4_ particles with good magnetothermal stability. In aqueous solution, the ethoxyl group (-C_2_H_5_O) contained in APTES will be hydrolyzed and transformed into silicon hydroxyl group (-Si-OH). And regarding nano Fe_3_O_4_@SiO_2_, the silicon hydroxyl group on the surface is obtained by dehydration condensation. Fe_3_O_4_@SiO_2_-(CH_2_)_3_-NH_2_ (hereinafter referred to as FSON) obtains a terminal-CH_2_-NH_2_ group, which can form FS-CH_2_-N-(CH_2_-CH_2_-COOCH_3_)_2_ by Michael addition with methyl acrylate, hereinafter referred to as FSNM. The synthesis route is shown in Figure 1.

The -Si-O-Si- structure formed by TEOS hydrolysis contains abundant silanol groups on the surface, which provide active sites for subsequent surface modification. A total of 5 g of nano-sized Fe_3_O_4_ was dispersed in 500 mL of 80% (*v*/*v*) ethanol aqueous solution by ultrasonic treatment for 20 min. The dispersion was transferred into a 1 L three-necked flask and stirred at 25 °C. Then, 10 mL of ammonia water (25 wt%) was added as a catalyst, followed by dropwise addition of 14 mL of TEOS over 30 min. The reaction was allowed to proceed for 6 h at 25 °C with constant stirring. Clean the residual TEOS and ammonia with anhydrous ethanol, dry and crush to obtain Fe_3_O_4_@SiO_2_ nanoparticles. The magnetic washing purification method actively screened the products with excellent magnetic response ability, but the particle size of the magnetic core is larger, and the multi-core Fe_3_O_4_ coated with SiO_2_ has a better magnetic response and is easier to settle, which can be confirmed in the image of the transmission electron microscope.

### 2.3. Surface Pretreatment of Fe_3_O_4_@SiO_2_ Particles

Before synthesizing FSON, Fe_3_O_4_@SiO_2_ particles need to be pretreated to change the distribution of silicon hydroxyl groups on the surface of the product to narrow the number distribution and indirectly control the number distribution of amine groups on the surface of the FSON branched core. The density of silicon hydroxyl groups on the surface of SiO_2_ prepared at 25 °C is about 4.6 n/Nm^2^, that is, about 7.6 × 10^−10^ mol·cm^2^. All three kinds of silicon hydroxyl groups can react with silane, and the reaction activity of ortho silicon hydroxyl groups is the strongest. Preparing silicon dioxide coated iron oxide nanoparticles Fe_3_O_4_@SiO_2_: After cleaning, drying and vacuum drying at 200 °C, the density of silicon hydroxyl groups on the treated surface was measured to be about 4.2 n/Nm^2^, that is, about 6.98 × 10^−10^ mol·cm^−2^. In this state, the main silicon hydroxyl groups on the surface were highly active ortho hydroxyl groups and slightly less active para hydroxyl groups. The grafting rate with APTES could exceed 40%.

### 2.4. Synthesis of Magnetic Polyamide Hyperbranched Molecules

In the following codes, N_1_ is the branched product of ethylenediamine, N_2_ is the branched product of 1,3-propanediamine, N_3_ is the branched product of diethylenetriamine, N_4_ is the branched product of triethylenetetramine, and N_5_ is the branched product of tetraethylenetriamine.

**Figure 2:**
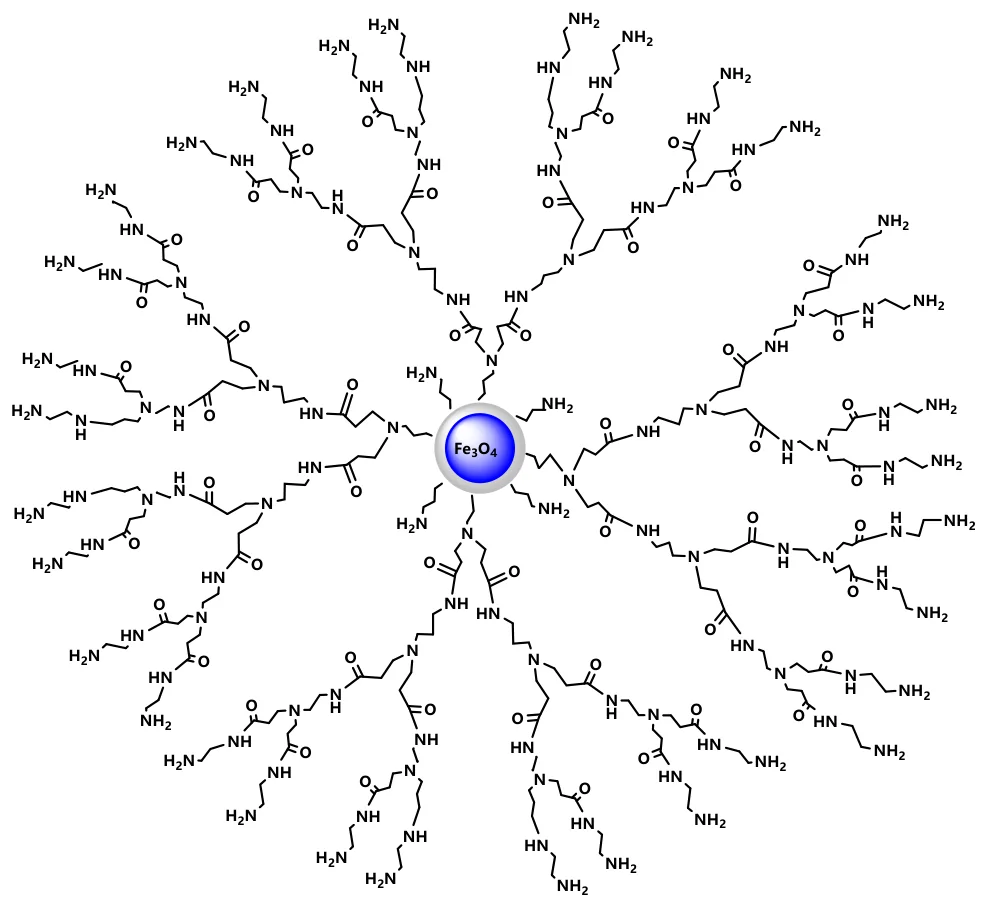
Hyperbranched molecules formed by hyperbranching of ethylenediamine.

Take 10 g of FSNM and dissolve it in 30 g of methanol. Use a constant pressure funnel to add saturated polyamine at a constant rate within 10 min. The molar ratio of polyamine to ester group in FSNM is 4:1. Keep the reaction at 50 °C for 12 h. FSNM will undergo the amidation reaction of esters to obtain the first generation of branched molecule 1-FSMN. Repeat the Michael addition reaction of synthesizing FSNM and amidation reaction of ester in this section. In the Michael addition reaction, acrylic acid is saturated, and the molar ratio of dosage to ethylenediamine is 4:1; the molar ratio of methyl methacrylate to amino groups is 2:1. Other conditions remain unchanged, and the second generation hyperbranched molecule (2-FSNMN) can be obtained. The hyperbranched molecules (3-FSNMN, 4-FSNMN, etc.) can be obtained by the Michael addition reaction and amidation reaction of repeated esters. For example, the hyperbranched molecule of ethylenediamine is shown in Figure 2. After the optimization of amine number and yield, it was found that the hyperbranched FSNMN_4_ molecule of triethylenetetramine had the highest amine number and higher yield: the 3-FSNMN_4_ molecule has the highest amine value (4.2 mmol/g) and a yield of up to 82.3%.

### 2.5. Synthesis of Cationic Polymer

A cationic polymer CAMHA with flocculating ability was synthesized with acrylamide (AM), acryloxyethyl trimethylammonium chloride (DAC) and maleic anhydride (MA). Ma was grafted into the long chain of the polymer. The polymerization chain growth stage of AM-DAC is an exothermic reaction. Under the condition of aqueous solution and constant temperature of 50 °C, the polymerization time is long, which can limit the polymerization rate to a controllable degree. The peak average molecular weight was determined by GPC method. The molecular weight of AM-DAC can reach about 4 million after 4–5 h of reaction, and more than 6 million after 7–8 h of reaction.

**Figure 3:**
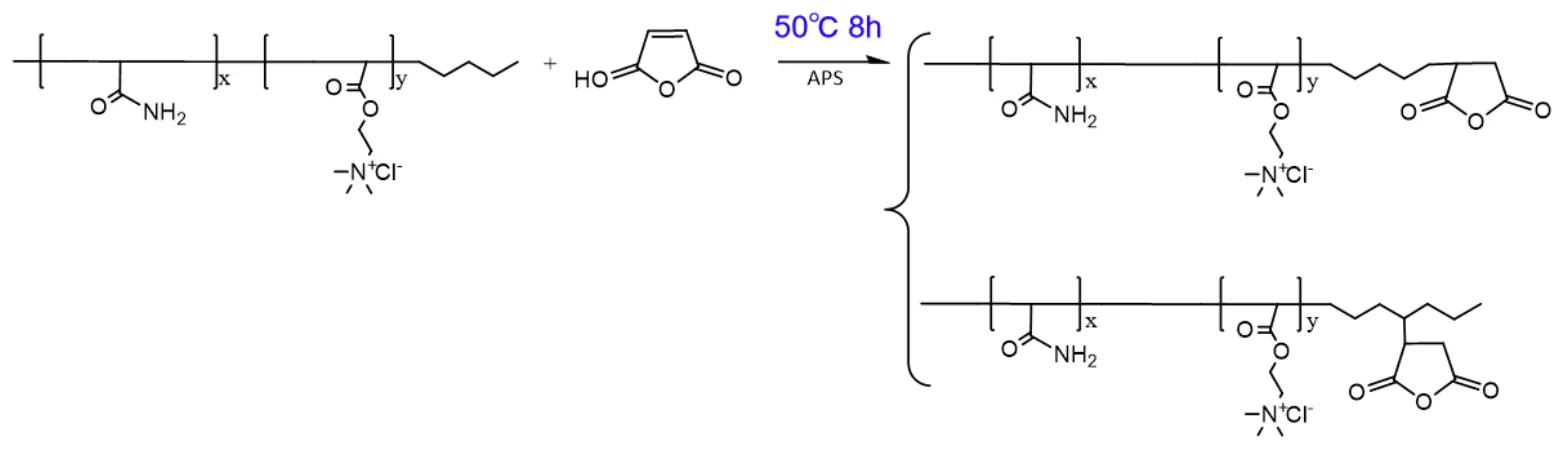
Synthesis of cationic polymer CAMHA.

Maleic anhydride monomer can react with polyolefin chain in polar solvent by initiator to obtain the graft product. The mainstream view is that the primary free radicals generated by the decomposition of the peroxide initiator attack the more active sites of the macromolecular chain to produce macromolecular free radicals, which are grafted with maleic anhydride through coupling and disproportionation termination. The degree of the grafting by solution method is about 1%. The reaction time was controlled and maleic anhydride was added to complete the grafting. The principle of synthesis is shown in Figure 3.

### 2.6. Synthesis of Magnetic Hyperbranched Cationic Polymer FSNMN-C

**Figure 4:**
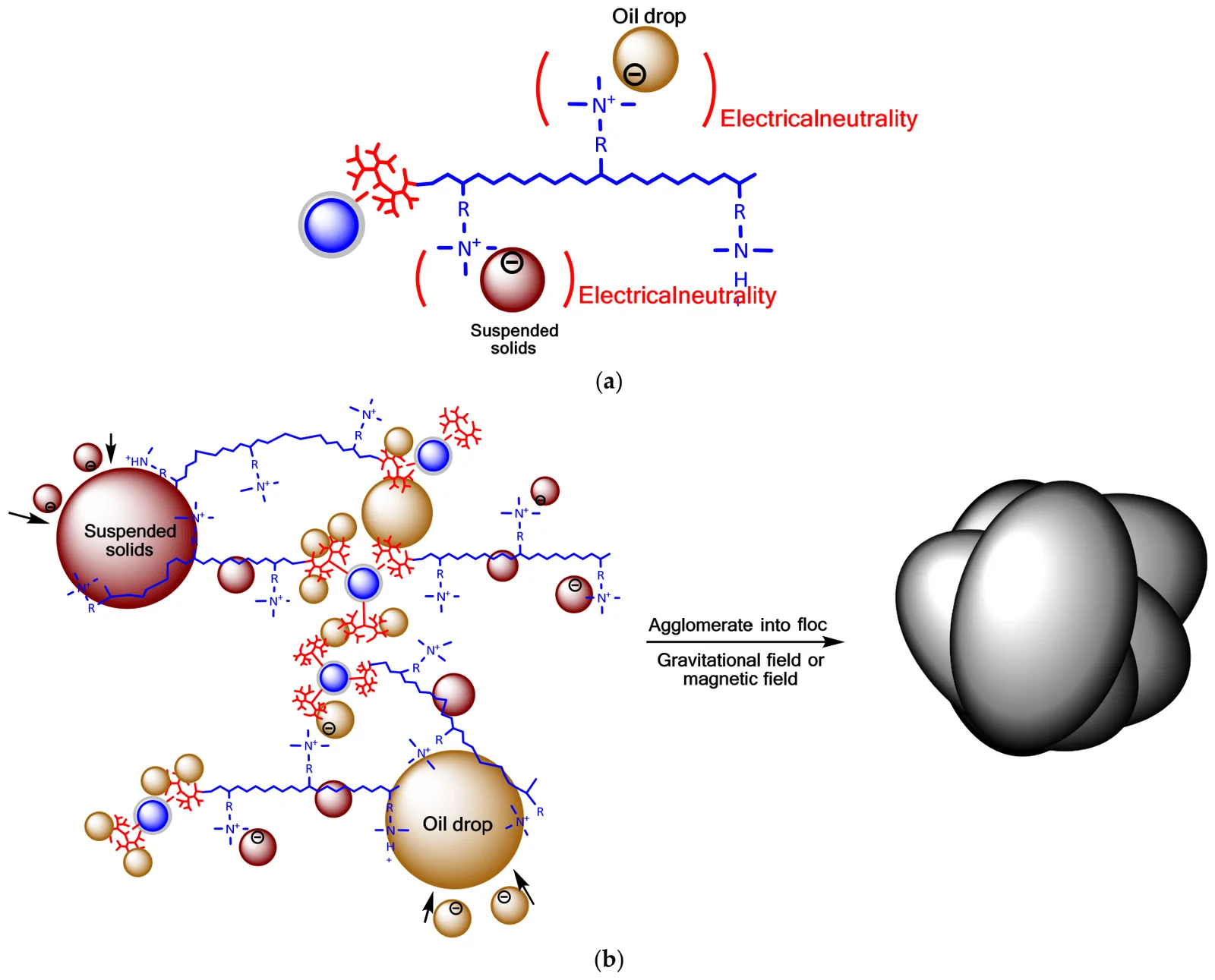
(**a**) Magnetic hyperbranched cationic polymer has electric neutralization; (**b**) Aggregation, netting, and flocculation of FSNMN-C.

The material (CAMHA) has a branched chain cationic long-chain structure (Figure 4), which can be forced to migrate by magnetic force in a magnetic field.

**Figure 5:**
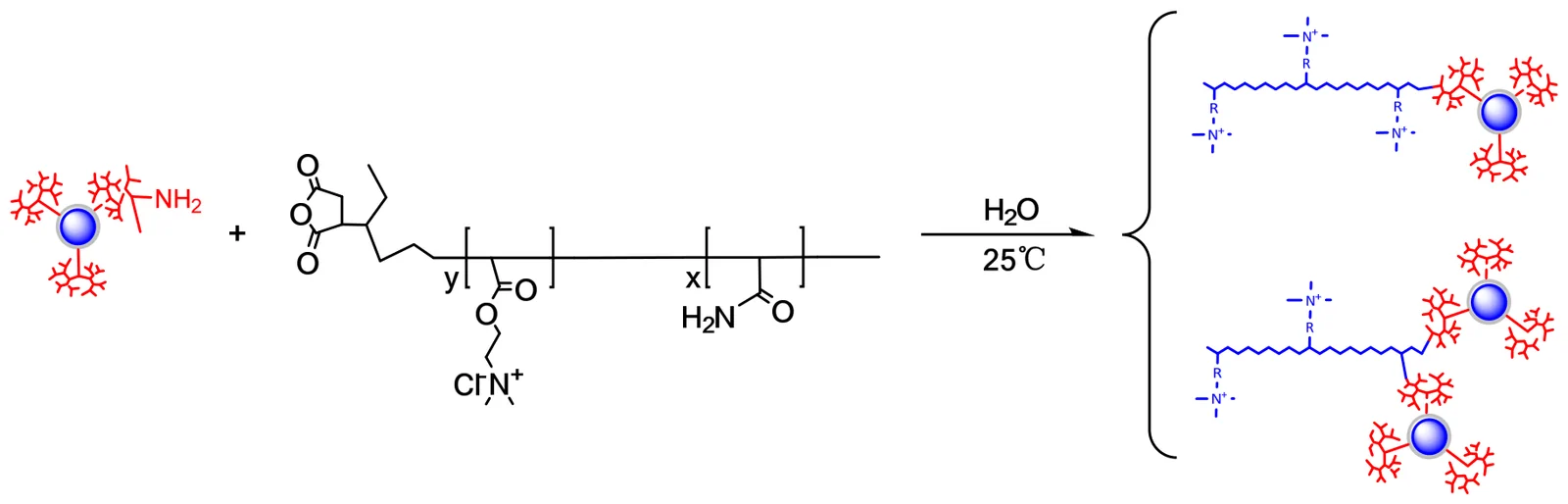
Grafting of hyperbranched molecular terminal amine groups onto cationic polymers.

The ring opening graft reaction between the hyperbranched molecular end amino group and maleic anhydride of the polymer chain can occur. Hyperbranched polymer products contain primary amine groups at the end, which can be linked with cationic long-chain maleic anhydride to form secondary amide groups and complete chemical connection, as shown in Figure 5.

The response surface methodology (RSM) experiment was designed with the removal rate of suspended solids as the response value, reaction temperature, reaction time, and reactant ratio (FSNMN:CAMHA). The experiment verifies and fits the model to obtain the optimal group, as shown in Table 1.

## 3. Results

The results showed that the optimal group was reaction time 4 h, reaction temperature 40 °C and reactant molar ratio 1:1, and the reactant FSNMN could be appropriately excessive. At this time, the removal rates of the five hyperbranched molecular grafted suspensions were 84.5%, 90.5%, 92.9%, 94.8% and 94.5%, respectively.

### 3.1. Structural and Morphological Characterization

(1) Fourier transform infrared spectroscopy (FT-IR) analysis

**Figure 6:**
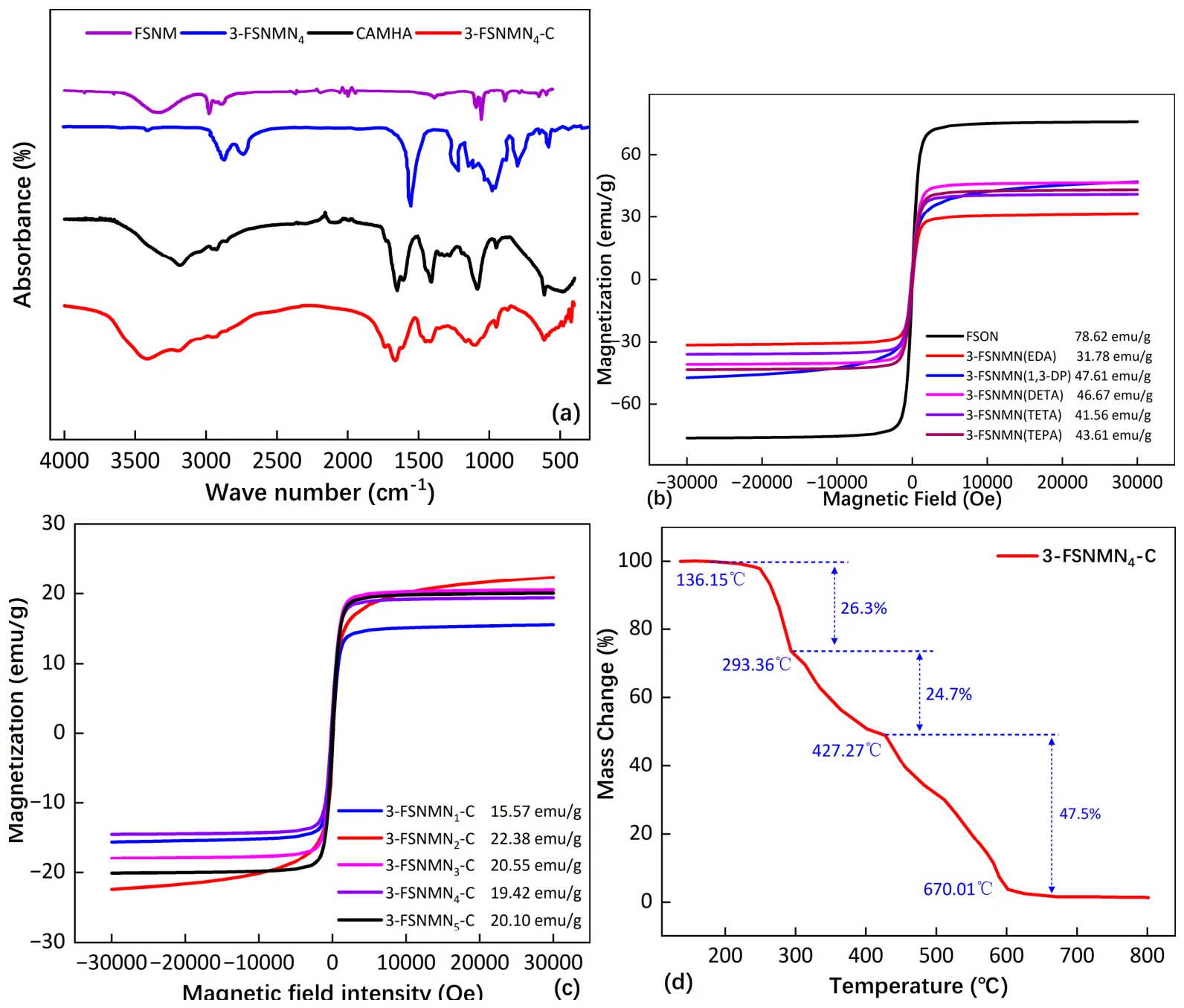
Infrared spectrum (**a**), magnetic response (**b**,**c**) and thermogravimetric analysis (**d**) of each product.

The optimal products FSNM,3-FSNMN_4_, CAMHA and 3-FSNMN_4_-C were used as infrared samples, and the samples were processed by KBr compression. The FT-IR spectra are shown in Figure 6.

Figure 6a shows that 3-FSNMN-C, 3-FSNMN and FSNM have similar characteristic structures. Compared with a FSNM molecule, 3-FSNMN-C has a significant amide absorption peak and branched structure. Compared with the characteristic line of 3-FSNMN, the characteristic peak is consistent with 3-FSNMN. There are strong and wide absorption peaks at 3346 cm^−1^, corresponding to the stretching vibration of N-H, amide absorption peak of secondary amide and -NH_2_ symmetric stretching vibration absorption peak. There were several methyl/methylene saturated carbon chain peaks in the range of 2973–2892 cm^−1^, of which the CH_3_ antisymmetric stretching vibration absorption peak appeared at 2973 cm^−1^ and the -CH_2_ antisymmetric stretching vibration absorption peak appeared at 2922 cm^−1^. There are multiple stretching vibration absorption peaks with C=O between 1934 and 2042 cm^−1^; 1378 cm^−1^ is the weak symmetrical vibrational absorption peak of -COO- [22]. It can be found that 3-FSNMN-C has a large number of amide structures compared with CAMHA spectral lines. After observing the contrast of other characteristic peaks, there are multiple overlapping spectral bands and absorption peaks in the range of wave numbers from 3500 cm^−1^ to 3000 cm^−1^. The moderate intensity symmetric stretching and antisymmetric stretching vibration peaks of amide-NH_2_ exist at the wave number of 3425 cm^−1^, which are superimposed with the dispersion absorption bands of ammonium salts, so -NH_2_ and NH_4_^+^structures may exist at the same time. The strong and sharp symmetric stretching vibration peaks of amide-HN_2_ appear at the wave number of 3182 cm^−1^, and the dispersion strong absorption band of ammonium salt NH_4_^+^is superimposed at the lower part of the band, while the moderate intensity and wide band of vibration appear at the wave number of 3037 cm^−1^, which conforms to the antisymmetric stretching band of NH_4_^+^. It is determined that there are amide structures and ammonium salt NH_4_^+^structures in the polymer molecules. Multiple stretching vibration peaks of -CH_2_-, -CH_3_ appeared near 2924 cm^−1^, which was also the antisymmetric stretching vibration characteristic peak of long-chain -CH_2_-. At 2850 cm^−1^, there was a symmetric stretching vibration peak of long-chain-CH_2_-. It was determined that the product was a saturated long carbon chain polymer. At 1732 cm^−1^, there was a low intensity absorption peak, a weak characteristic five-membered ring C=O stretching vibration absorption peak at this position, and a very strong carbonyl stretching vibration peak at 1651 cm^−1^, while carbonyl existed in both amide and -COO- structures, with a strong antisymmetric stretching vibration peak of carboxyl -COO- at 1608 cm^−1^, a carboxyl -COO- symmetric stretching vibration peak at 1408 cm^−1^, and a stretching vibration absorption peak of aliphatic anhydride at 1083 cm^−1^. It was determined that there was an anhydride structure, which was weaker than CAMHA, indicating that the grafting process consumed part of the anhydride structure.

By comparing and analyzing the infrared spectra, it can be seen that the infrared characteristics of 3-FSNMN-C include all the characteristic groups of hyperbranched and cationic chains, indicating that there are hyperbranched molecules and cationic polymer structures in the 3-FSNMN-C molecule at the same time.

(2) Analysis of vibrating magnetometer

The saturation magnetization curve is measured by vibrating sample magnetometer (VSM), which is used to test the magnetic properties of magnetic materials. The magnetization curve is tested and drawn with a Lake Shore 7410 vibrating sample magnetometer, as shown in Figure 6.

Figure 6b shows the saturation magnetization of FSNMN before grafting. The initial magnetic core is 78.62 emu/g. When branched to the third-generation hyperbranched molecule, the 3-FSNMN_1_ hyperbranched ethylenediamine is 31.78 emu/g, the 3-FSNMN_2_ hyperbranched of 1,3-propanediamine is 47.61 emu/g, the 3-FSNMN_3_ hyperbranched of diethylenetriamine is 33.56 emu/g, the 3-FSNMN_4_ hyperbranched of triethylenetetramine is 41.56 emu/g, and the 3-FSNMN_5_ hyperbranched of tetraethylenetriamine is 41.56 emu/g.43.61 emu/g. The magnetic responsiveness of the products are lower than 90 emu/g of nano-Fe_3_O_4_ particles and much higher than 21 emu/g of natural ferrite particles. The hysteresis loop curve passes through the origin without a hysteresis phenomenon, and can quickly respond to the magnetic force of the magnetic field to produce displacement, and has sufficient magnetic responsiveness.

Figure 6c shows that the magnetic response ability of the grafted FSNMN-C is attenuated to a certain extent, and the saturated magnetization of the grafted FSNMN-C is reduced to varying degrees, possibly due to the shielding effect of the thicker polymer chain on the magnetic field. The hyperbranched 3-FSNMN_1_-C of ethylenediamine was 15.57 emu/g, the hyperbranched 3-FSNMN_2_-C of 1,3-propanediamine was 22.38 emu/g, the hyperbranched 3-FSNMN_3_-C of diethylenetriamine was 20.55 emu/g, the hyperbranched 3-FSNMN_4_-C of triethylenetetramine was 19.42 emu/g, and the hyperbranched 3-FSNMN_5_-C of tetraethylenetriamine was 20.10 emu/g. Although the magnetic response ability is weakened, it is similar to the 21 emu/g of natural ferrite particles. The hysteresis loop curve passes through the origin without a hysteresis phenomenon, and can quickly respond to the magnetic force of the magnetic field and shift.

(3) Thermogravimetric analysis of FSNMN-C

The instrument uses a DSC-Q2000 differential scanning calorimeter of TA company in the United States. The experimental sample is 3-FSNMN_4_-C hyperbranched grafted with triethylenetetramine. The thermal decomposition curve is shown in Figure 6d.

After vacuum drying, the water of the sample was completely lost at 136.15 °C, indicating that there was bound water in its molecules. The thermogravimetry curve after moisture removal generally presents a three-stage pattern. Due to the hyperbranched structure with long chains and multiple branches, the ladder of the curve is not obvious. 3-FSNMN_4_-C began to decompose at a high speed at 249.13 °C, and the slope of the mass change curve with temperature was the largest, and it could maintain thermal stability at 136.15–249.13 °C.

In the first stage, from 249.13 °C to 293.36 °C, the mass loss was mainly caused by thermal imidization of the amide structure and thermal decomposition of the quaternary ammonium structure, with a total mass loss of 26.3%. In the second stage, from 296.1 °C to 409.1 °C, the whole polymer chain began to decompose, the main chain was broken by heat, and the slope of the curve was high. The maleic anhydride group also began to decompose at 330 °C, with a loss of 24.7% of the total mass. The third stage is from 427.27 °C to 670.01 °C, which is the final stage of decomposition of the products and the remaining heat-resistant groups. With the continuous trend of decomposition slowing down, the thermal decomposition is completed after 670.01 °C, with a total loss of 47.5% of the total mass. Up to 800 °C, a certain quality of magnetic core remains and does not decompose. The thermogravimetric curve is similar to the decomposition curve of CAMHA, and has unique decomposition characteristics. The thermogravimetric thermal property is consistent with the structure of FSNMN-C hyperbranched cationic polymer.

(4) Scanning electron microscope analysis of FSNMN-C

**Figure 7:**
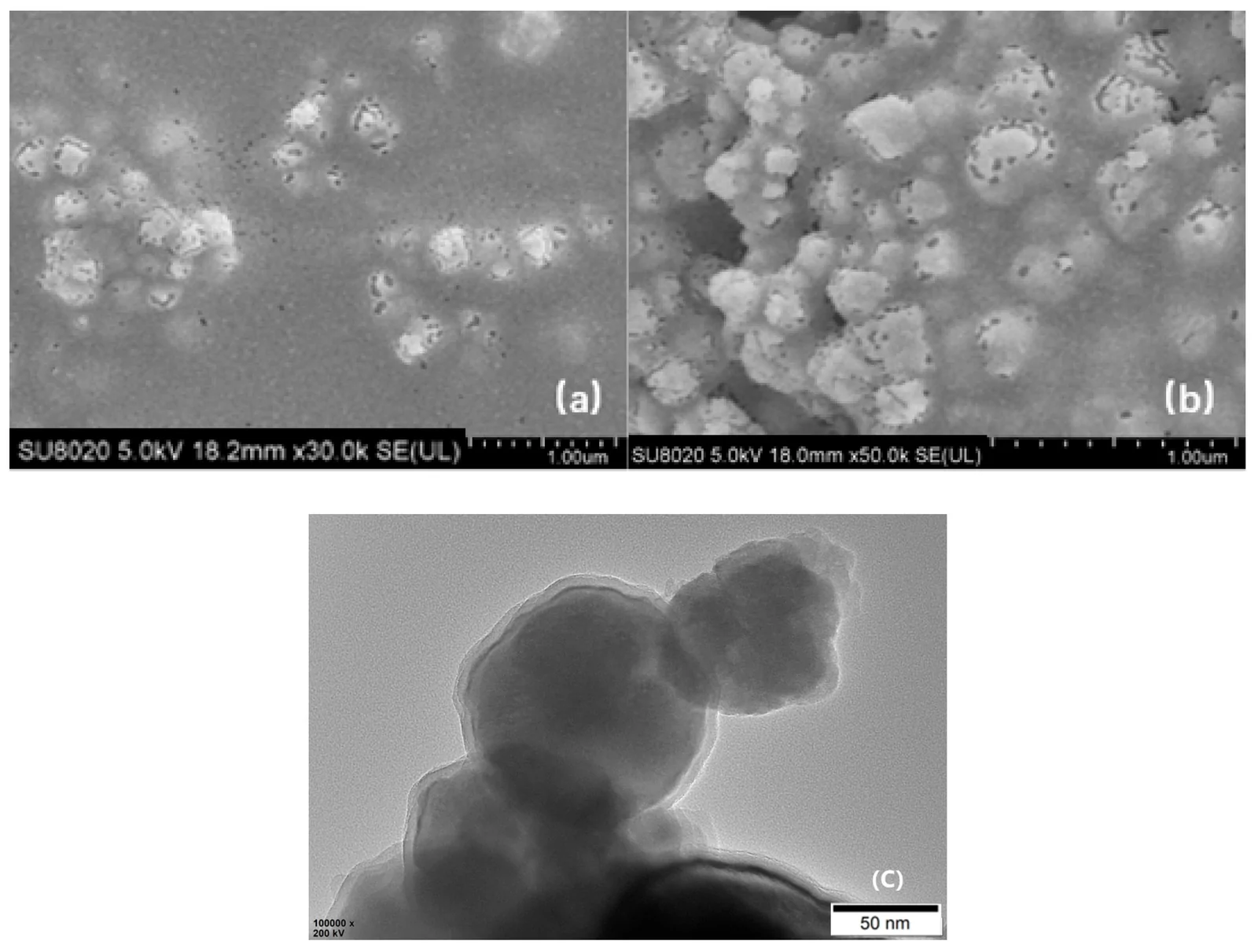
(**a**,**b**) SEM images of 3-FSNMN-C, (**c**) TEM image of 3-FSNMN-C.

The scanning electron microscope (SEM) uses the Hitachi su8020 (EMAXevolution X-Max80/EX-270) magnetic material scanning electron microscope, manufactured by Hitachi High-Technologies Corporation, Tokyo, Japan. The electron microscope sample is the third-generation triethylenetetramine hyperbranched and grafted 3-FSNMN_4_-C. The sample is dissolved and dried into thin sheets for SEM scanning electron microscope imaging. The sample is a non-conductive organic matter with poor electron beam imaging effect, so it needs to be sprayed with gold. The SEM and TEM images are shown in Figure 7.

Figure 7a shows that the material after drying and shrinkage of 3-FSNMN_4_-C has an obvious hyperbranched molecular core, and the shape and size of the core match that in Section 2. The sphere is a complete hyperbranched molecule, and the surface of the material is obviously covered with organic matter. Hyperbranched molecules are evenly distributed in a large range in the 3-FSNMN_4_-C dry material. It is found in Figure 7b that they are clustered and distributed in a small range after magnification, thus maintaining their magnetic response performance. The TEM image in Figure 7c shows that the surface of the iron core was successfully coated with a silica shell. The multi-stage maleic anhydride groups generated in the second stage of CAMHA synthesis polymerization can be grafted with multiple hyperbranched molecules, which also conforms to the reaction mode of CAMHA dropping into FSNMN.

### 3.2. Dispersion Performance and Cationic Degree of FSNMN-C

The laser observation method was used to verify the dispersion performance of each molecule in water. When the average molecular weight is 4 million, the dispersion time is almost the same, and the time difference is very small.

Magnetic hyperbranched cationic linear chain molecules contain magnetic nuclei with relatively concentrated density, so the molecular weight has a great impact on the static stability of the solution. The static stability of FSNMN-C molecules with the same mass dispersed in water is evaluated. The dispersion stability of 3-FSNMN-C is good, and the precipitation mass after standing for 10 days is less than 1%. The average molecular weight of 3-FSNMN obtained under the optimized synthesis conditions is 4 million~5 million, while the dispersion of 3-FSNMN_4_ and 3-FSNMN_5_ is stable without precipitation.

The cationic degree of FSNMN-C material was determined by silver nitrate potassium dichromate titration. Table 2 shows that the cationic degree of grafted 3-FSNMN-C material can be maintained above 20%, and the cationic degree of 3-FSNMN_4_-C synthesized under the optimal conditions is 20.5%.

### 3.3. Demulsification Performance of FSNMN-C

The simulated emulsion has stable properties, which can control variables and comparative analysis. The demulsification performance of different hyperbranched molecules is evaluated by using the simulated emulsion, and data is accumulated for mechanism analysis. Using standard kerosene, sodium dodecylbenzene sulfonate, and sodium petroleum sulfonate as the solvent, hydrolyze anionic polyacrylamide HPAM, an emulsion with oil content of 1000 mg/L and zeta potential of −35 mV was prepared, which is recorded as negative emulsion a-; using standard kerosene, Tween span and non-ionic polyacrylamide non-PAM, an emulsion with oil content of 1000 mg/L and an electric neutral kerosene emulsion were prepared, which is recorded as electric neutral emulsion a; using standard kerosene, bentonite, superfine water-borne calcium carbonate, sodium dodecylbenzene sulfonate, sodium petroleum sulfonate, polyanionic cellulose sodium and hydrolyzed anionic polyacrylamide HPAM to prepare an emulsion with oil content of 1000 mg/L, the kerosene emulsion of negatively charged suspended matter with oil content of 1000 mg/L, suspended matter of 150 mg/L and zeta potential of −35 MV was prepared.

The demulsification properties of two kinds of simulated emulsions, negatively charged emulsion a- and electrically neutral emulsion a, were evaluated. Adjust the dosage of the treatment agent, set the experimental temperature as 25 °C, shake well with a plug, during which the plug can be opened multiple times to exhaust, shake well and then stand for 30 min. The centrifugal force field uses a centrifuge with a rotating speed of 3000 r/min, add the agent and mix well, centrifuge for 10 min and then stand for 20 min. Take the middle clear liquid to determine the oil content and calculate the oil removal rate. The effect of FSNMN-C on demulsification of the simulated liquid under the conditions of gravity field and magnetic field was compared and evaluated, and the centrifugal force field was the best control group.

#### 3.3.1. Demulsification of Electrically Neutral Emulsion by FSNMN-C

**Figure 8:**
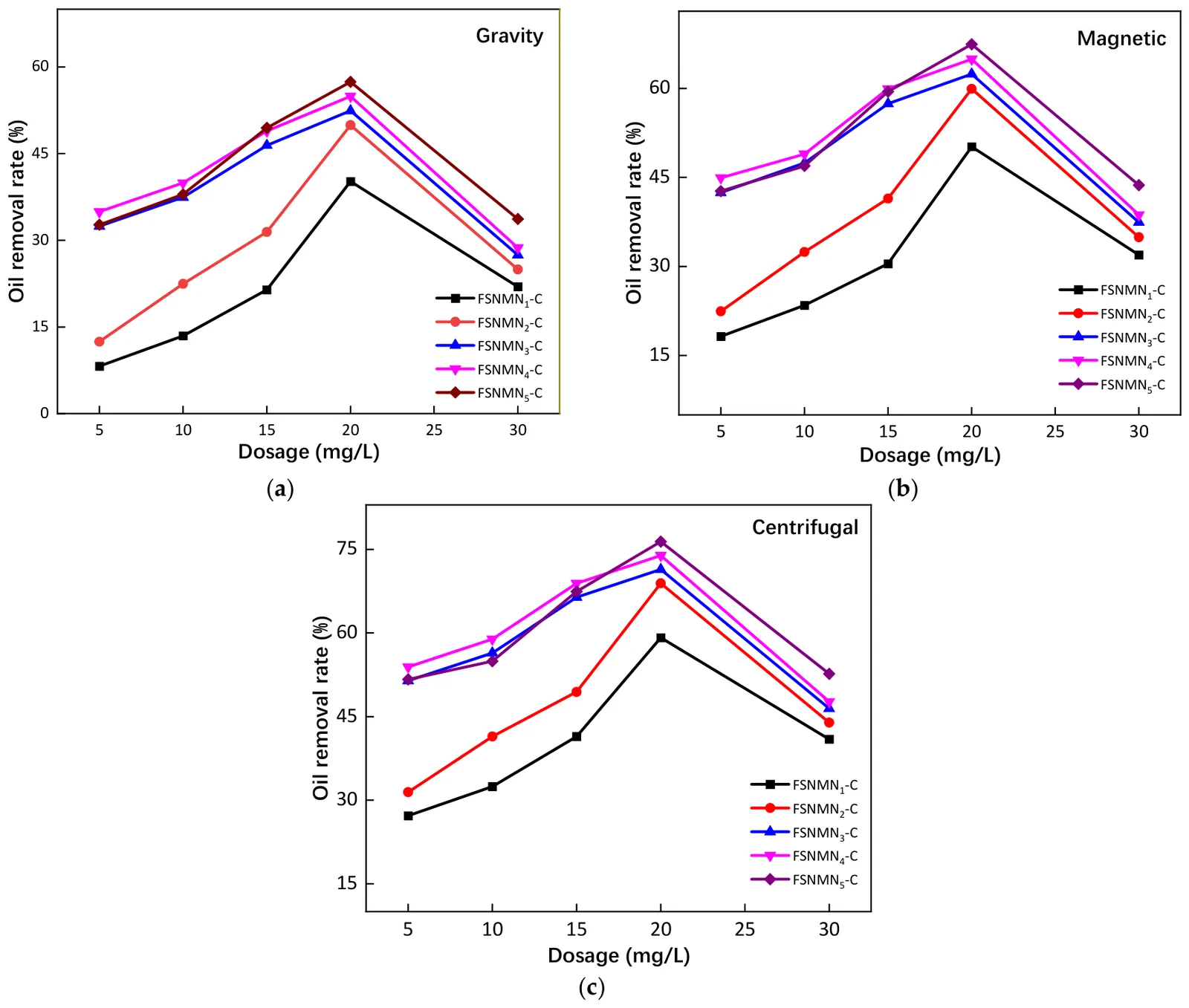
Demulsification experiment of 3-FSNMN-C electroneutral lotion, (**a**) Gravity subsidence, (**b**) magnetic field treatment, (**c**) centrifugal treatment.

The experimental results are shown in Figure 8.

It can be seen from Figure 8 that comparing the treatment effect of 3-FSNMN-C on electroneutral emulsion under different conditions, the optimal dosage is 20 mg/L, and the treatment effect under magnetic field is stronger than that of natural precipitation under gravity field, with 3-FSNMN_5_-C at 67.41% and 3-FSNMN_4_-C at 64.93%. In the same 30 min separation time, the demulsification effect is higher than that of natural precipitation under gravity field, with 3-FSNMN_5_-C at 57.42% and 3-FSNMN_4_-C at 54.93%. The treatment ability of the control group under centrifugal force field was the strongest: 3-FSNMN_5_-C was 76.31% and 3-FSNMN_4_-C was 73.89%.

It can be seen that 3-FSNMN-C has the treatment ability for electroneutral emulsion, but the treatment effect will not exceed 76%.

#### 3.3.2. Demulsification of Negatively Charged Emulsion by FSNMN-C

**Figure 9:**
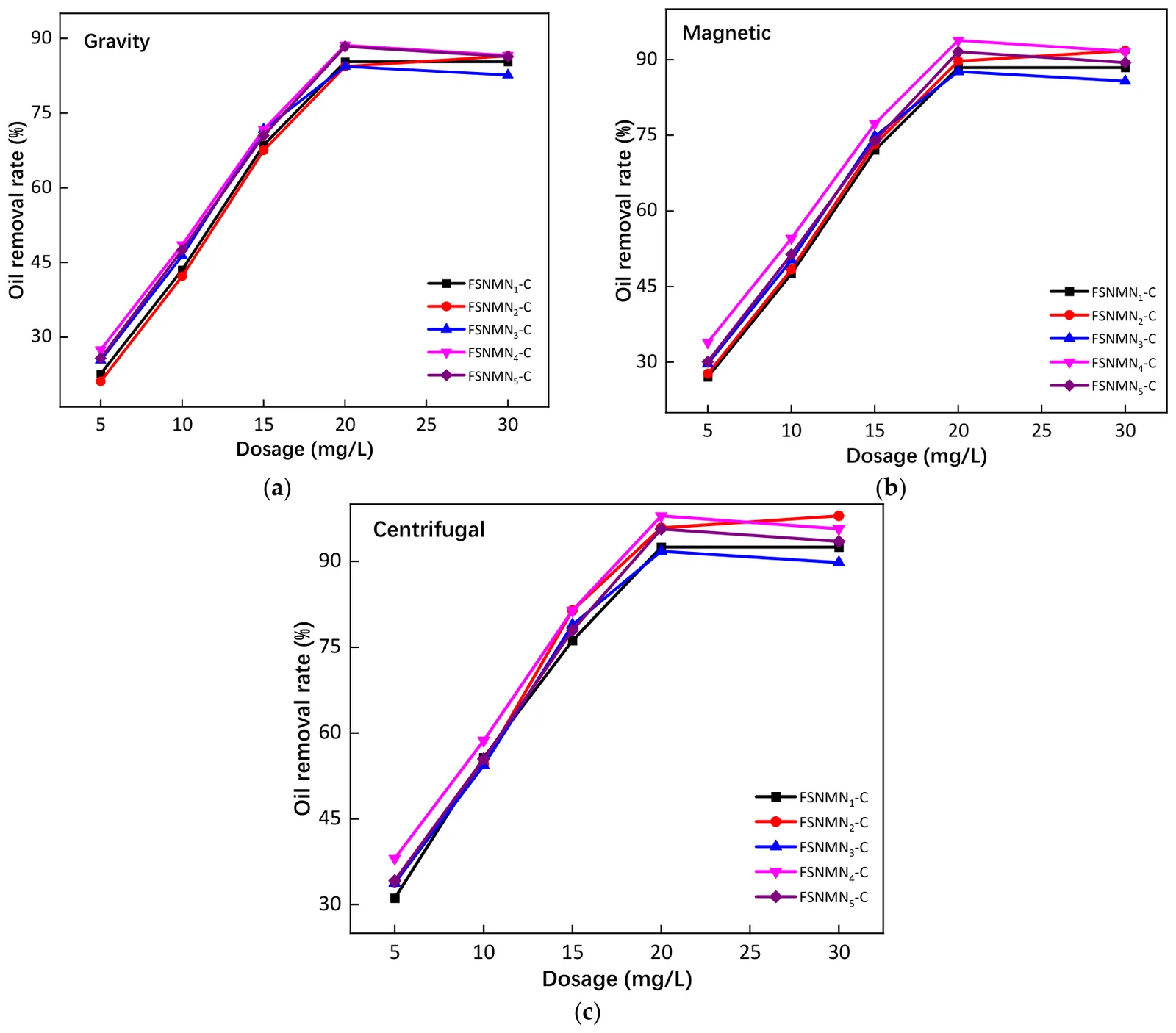
Demulsification experiment of 3-FSNMN-C negative electric lotion, (**a**) Gravity subsidence, (**b**) magnetic field treatment, (**c**) centrifugal treatment.

The experimental data are shown in Figure 9.

It can be seen from Figure 9 that the effect of 3-FSNMN-C on the treatment of negatively charged emulsion is stronger. Comparing the treatment effect of 3-FSNMN-C on negatively charged emulsion under different conditions, the optimal dosage is 20 mg/L. The treatment effect under magnetic field is stronger than that under gravity field. The treatment effect of 3-FSNMN_4_-C is 93.82%, and that of 3-FSNMN_5_-C is 91.55%. In the same 30 min separation time, it is higher than that under gravity field. The treatment effect of 3-FSNMN_5_-C and 3-FSNMN_4_-C is the same at 88.39%. The demulsification ability of the control group under centrifugal force field was the strongest, and the oil removal rate of 3-FSNMN_5_-C was the highest, 97.95%, followed by that of 3-FSNMN_4_-C, 95.68%.

It can be seen that the magnetic treatment effect of 3-FSNMN-C for the negatively charged emulsion is close to that of centrifugal treatment, and the oil removal rate is more than 94% under the condition of appropriate dosage. 3-FSNMN_4_-C and 3-FSNMN_5_-C have the same emulsion treatment ability and the strongest demulsification ability.

### 3.4. Flocculation Performance of FSNMN-C

Two kinds of simulated suspensions, negative charged suspension B- and electroneutral emulsion B, were used to evaluate the flocculation performance of the products. Adjust the dosage of the treatment agent, set the experimental temperature at 25 °C, shake well with a plug, during which the plug can be opened multiple times to exhaust, shake well and then stand for 30 min, take the middle clear liquid to measure the content of suspended solids, and calculate the removal rate of suspended solids. The effect of FSNMN-C on the flocculation effect of simulated liquid under the conditions of gravity field and magnetic field was compared and evaluated, and the centrifugal force field was the best control group.

#### 3.4.1. Flocculation Experiment of FSNMN-C for Electrically Neutral Suspension

**Figure 10:**
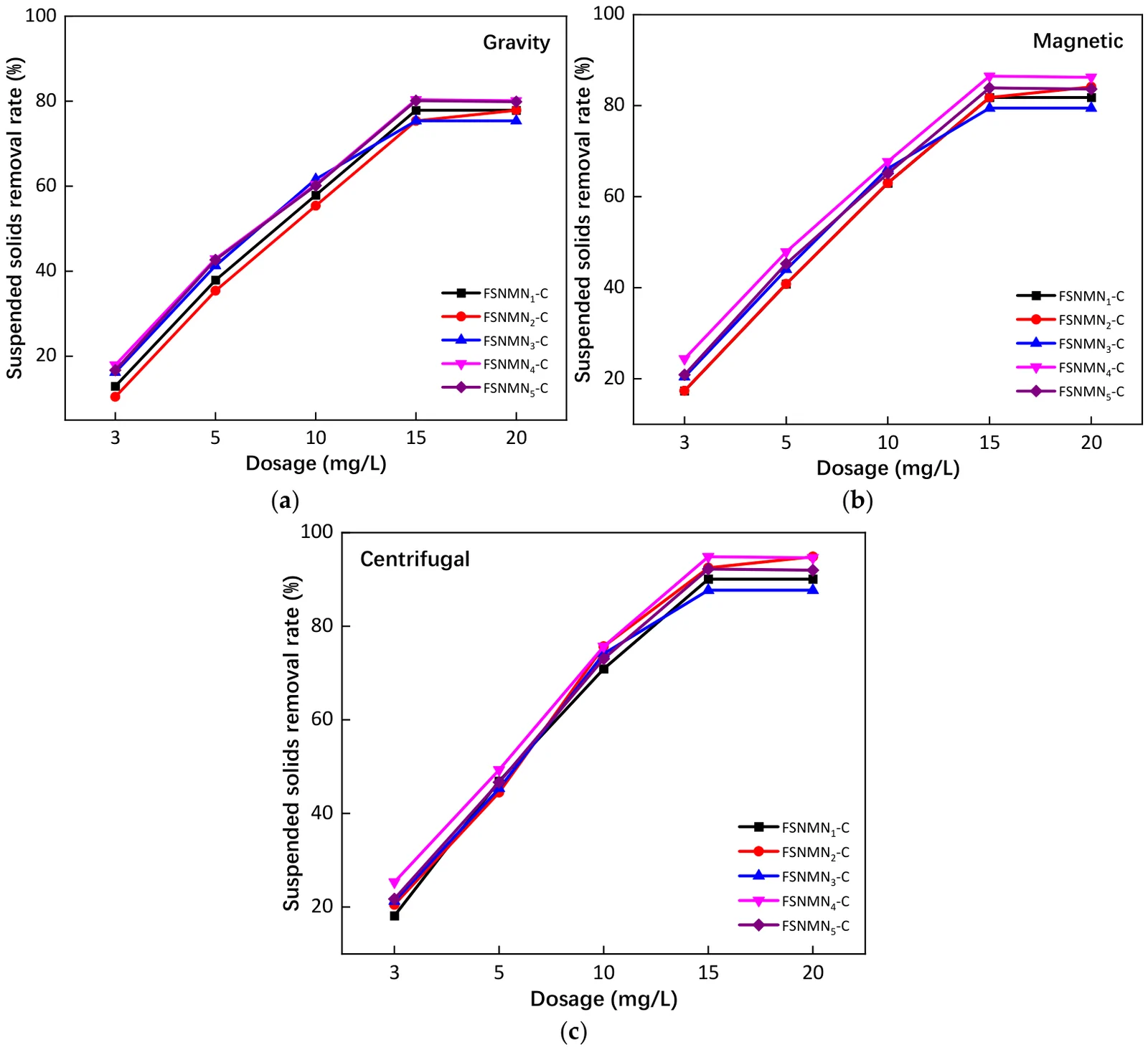
Flocculation experiment of 3-FSNMN-C neutral suspension, (**a**) Gravity subsidence, (**b**)magnetic field treatment, (**c**) centrifugal treatment.

The experimental data are shown in Figure 10.

By comparing the treatment effect of 3-FSNMN-C on electrically neutral suspensions under different conditions (Figure 10), the dosage was found to be 15 mg/L to achieve the optimal dosage. The treatment effect under magnetic field is stronger than that under gravity field. The 3-FSNMN_4_-C is 86.48%, and the 3-FSNMN_5_-C is 83.90%. In the same 30 min separation time, the additional magnetic field accelerates the settlement of flocs. The treatment ability of the control group under centrifugal force field is the strongest. The removal rate of suspended solids of 3-FSNMN_4_-C is 94.86%, and that of 3-FSNMN_5_-C is 92.23%.

It can be seen that 3-FSNMN-C has strong treatment capacity for electroneutral suspensions.

#### 3.4.2. Flocculation Experiment of Negative Charge Suspension by FSNMN-C

**Figure 11:**
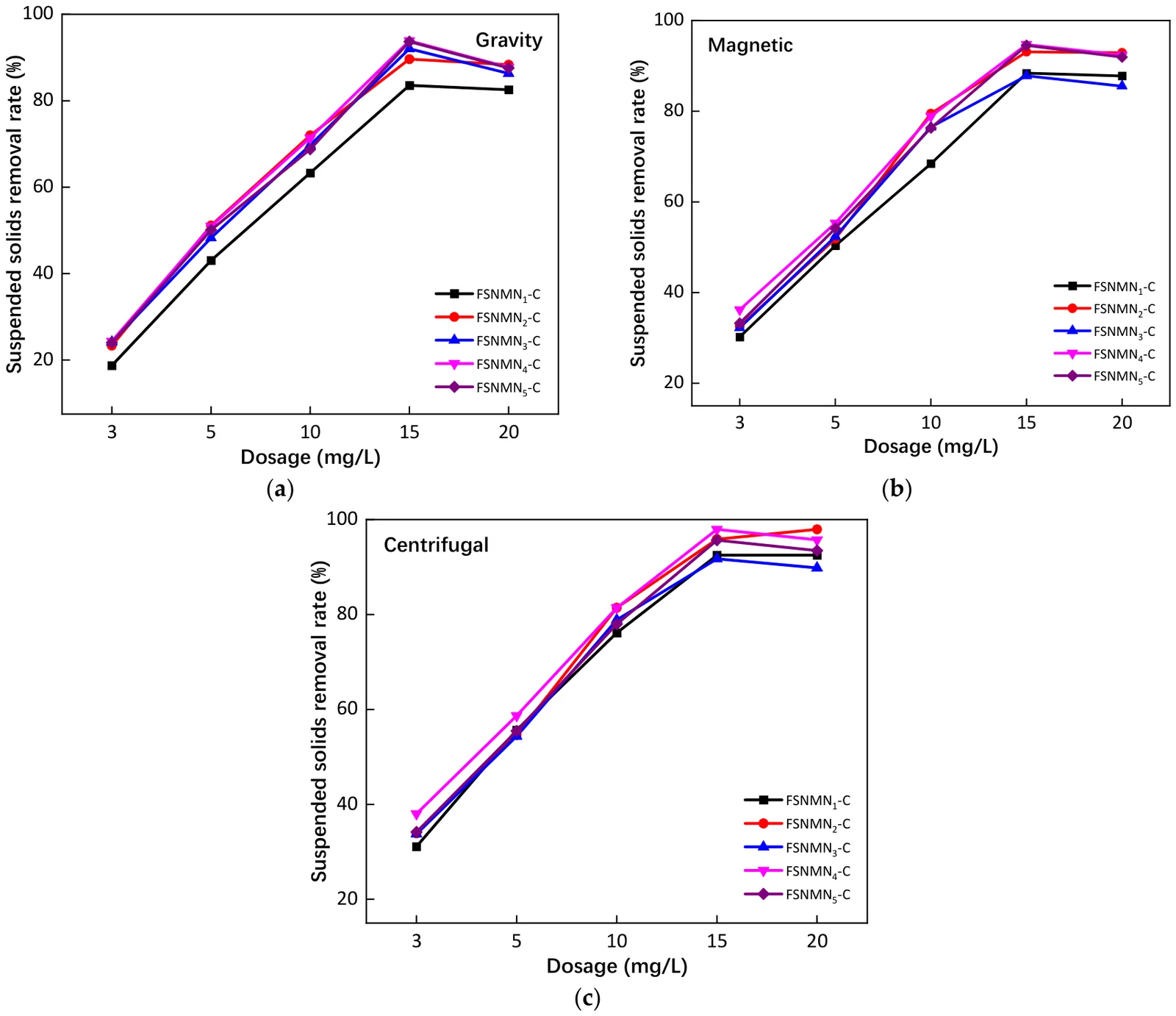
Flocculation experiment of 3-FSNMN-C negative suspension, (**a**) Gravity subsidence, (**b**) magnetic field treatment, (**c**) centrifugal treatment.

The experimental data are shown in Figure 11.

By comparing the treatment effect of 3-FSNMN-C on electrically neutral suspension under different conditions (Figure 11), the optimal dosage is 15 mg/L, and the treatment effect under magnetic field is also stronger than that under gravity field. The treatment effect of 3-FSNMN_4_-C and 3-FSNMN_5_-C is the same at 94.52%. In the same 30 min separation time, the additional magnetic field accelerates the settlement of flocs, and the treatment effect is also higher than that under gravity field. The gravity settlement is 93.67%. The treatment ability of the control group under centrifugal force field was the strongest: the removal rate of suspended solids of 3-FSNMN_4_-C was 97.95%, and that of 3-FSNMN_5_-C was 95.68%. It can be seen that 3-FSNMN-C has a strong treatment ability for negatively charged suspensions, and the effect of magnetic treatment is close to that of centrifugal treatment.

The experimental results show that 3-FSNMN-C has a strong ability to treat suspensions, and can effectively treat both electrically neutral and negatively charged suspensions. 3-FSNMN_4_-C and 3-FSNMN_5_-C have the strongest ability, and the best effect can be achieved when the suspended solids removal rate is 15 mg/L.

The yield of 3-FSNMN_4_-C is better than that of 3-FSNMN_5_-C, and the performance is close, so 3-FSNMN_4_-C is the best choice.

### 3.5. Performance of FSNMN in Treating ASP Flooding Wastewater

ASP flooding sewage sample: The separated sewage sample from a three-phase separation combined station in Daqing Oilfield has the following physical properties: suspended solids 143 mg/L, oil 921.09 mg/L, zeta potential −37 mV, pH 11. The magnetic field uses a self-made test tube rack group. The direction of the magnetic field is adjusted by the position of the permanent magnet. An N35 NdFeB permanent magnet is used. The maximum magnetic energy product is 35 MGOe, i.e., 270 Ka/m^3^; the magnetic field strength in the central area of the experimental setup, as measured by a teslameter, is 120 mT, with a magnetic field gradient of 15 T/m. The ferromagnetic core coated with a thin silica layer has a strong magnetic response and structural stability, which is more conducive to magnetic separation and recycling. The treatment efficiency of 88% can still be maintained after five times of reuse.

(1) Performance of FSNMN-c in Treating ASP Flooding Wastewater

**Figure 12:**
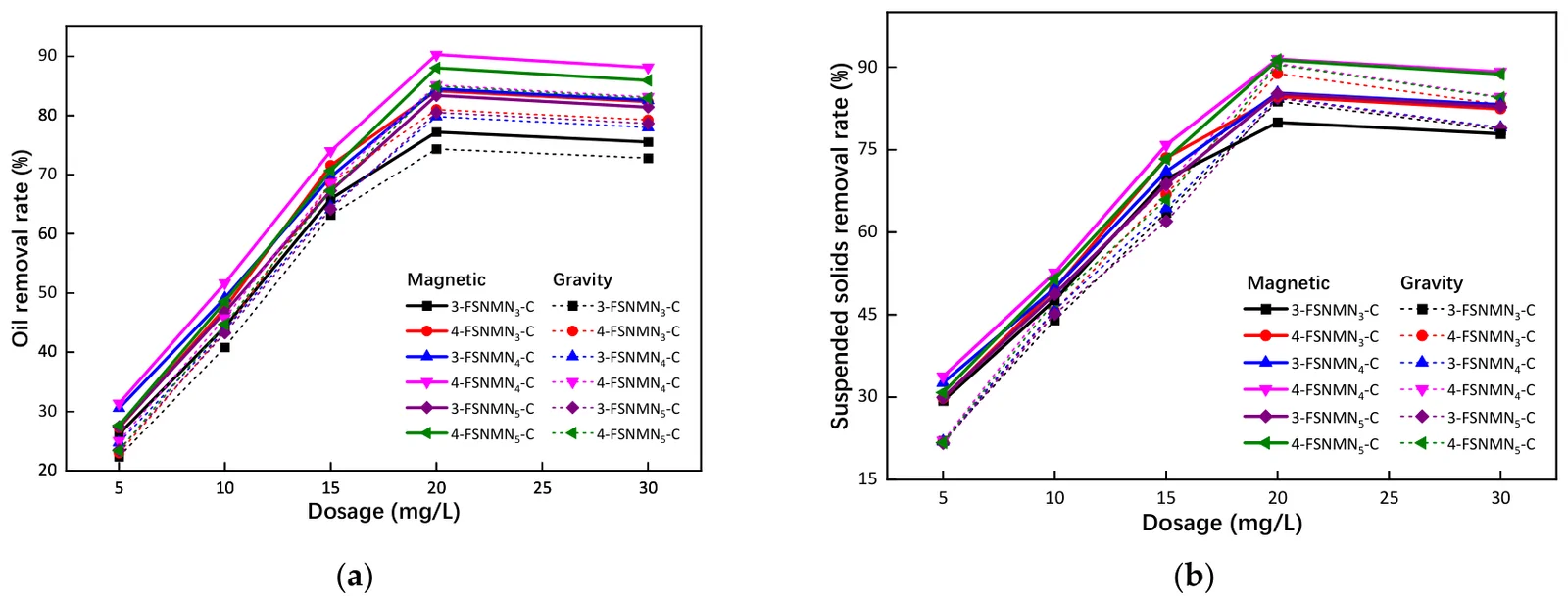

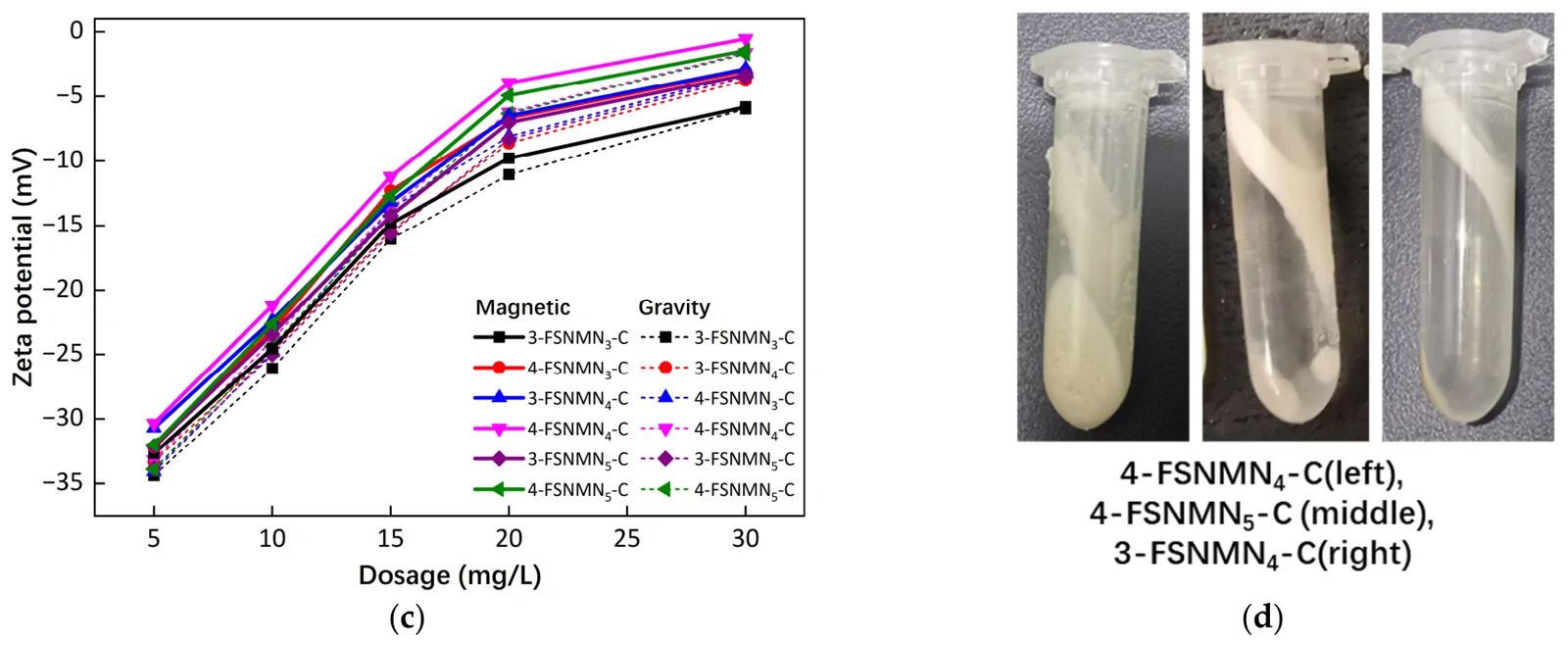
Performance of FSNMN-C in treating ASP flooding wastewater, (**a**) gravity subsidence, (**b**) magnetic field treatment, (**c**) centrifugal treatment, (**d**) after centrifugal treatment of wastewater.

Taking ASP flooding wastewater as the treatment object, the treatment performance of the material was evaluated. Adjust the dosage of the treatment agent, set the experimental temperature at 25 °C, shake well with a plug, during which the plug can be opened multiple times to exhaust, shake well and then stand for 30 min, take the middle clear liquid to determine the oil content, and calculate the oil removal rate. Under the optimal conditions, the yield of 3-FSNMN-C is better than that of 4-FSNMN-C, but the performance is similar. In this section, these two types of hyperbranched cationic polymers are used for comparative experiments. Compare and evaluate the effect of FSNMN-C on the demulsification effect of ASP flooding wastewater under the conditions of gravity field and magnetic field, sort out the experimental data, and get Figure 12.

Figure 12 shows that when the dosage is 20 mg/L, the demulsification effect of 4-FSNMN_4_-C and 4-FSNMN_5_-C under the condition of gravity field is the best, and the demulsification effect is almost the same, up to 85.13%, while the demulsification effect under the condition of magnetic field increases to 90.27% and 88.03%. The demulsification effect of the comprehensive optimization group 3-FSNMN_4_-C on ASP flooding wastewater under the condition of gravity field can reach 79.82%, while the demulsification effect under the condition of magnetic field can increase to 84.52%. When the dosage exceeds 20 mg/L, the demulsification effect decreases.

When the dosage was 20 mg/L, the flocculation effect of 4-FSNMN_4_-C and 4-FSNMN_5_-C under gravity field was the best, and the effect was almost the same; the removal rate of suspended solids reached 90.68%, while the flocculation effect under magnetic field was also the same, slightly increased to 91.32%. The flocculation effect of comprehensive optimization group 3-FSNMN_4_-C on ASP flooding wastewater under the condition of gravity field can reach 84.57%, while the flocculation effect under the condition of magnetic field can increase to 85.34%. When the dosage exceeds 20 mg/L, the flocculation effect decreases.

The zeta potential of ASP flooding wastewater used in the experiment is about −37 mV, and the particles are in a very stable state. When the dosage reached 20 mg/L, the zeta potential of the treated ASP flooding sewage particles basically decreased to −10 mV, and the stability of particle dispersion was poor, reaching the level of easy separation. The zeta potential of the particles treated with two kinds of 4-FSNMN-C decreased to −5 mV. It can be seen from the figure that with the increase in the dosage of magnetic hyperbranched FSNMN-c molecules, the absolute value of zeta potential decreased, and there was no electrical reversal within the dosage range of this experiment.

(2) Performance of magnetic treatment of ASP flooding wastewater by FSNMN-C

**Figure 13:**
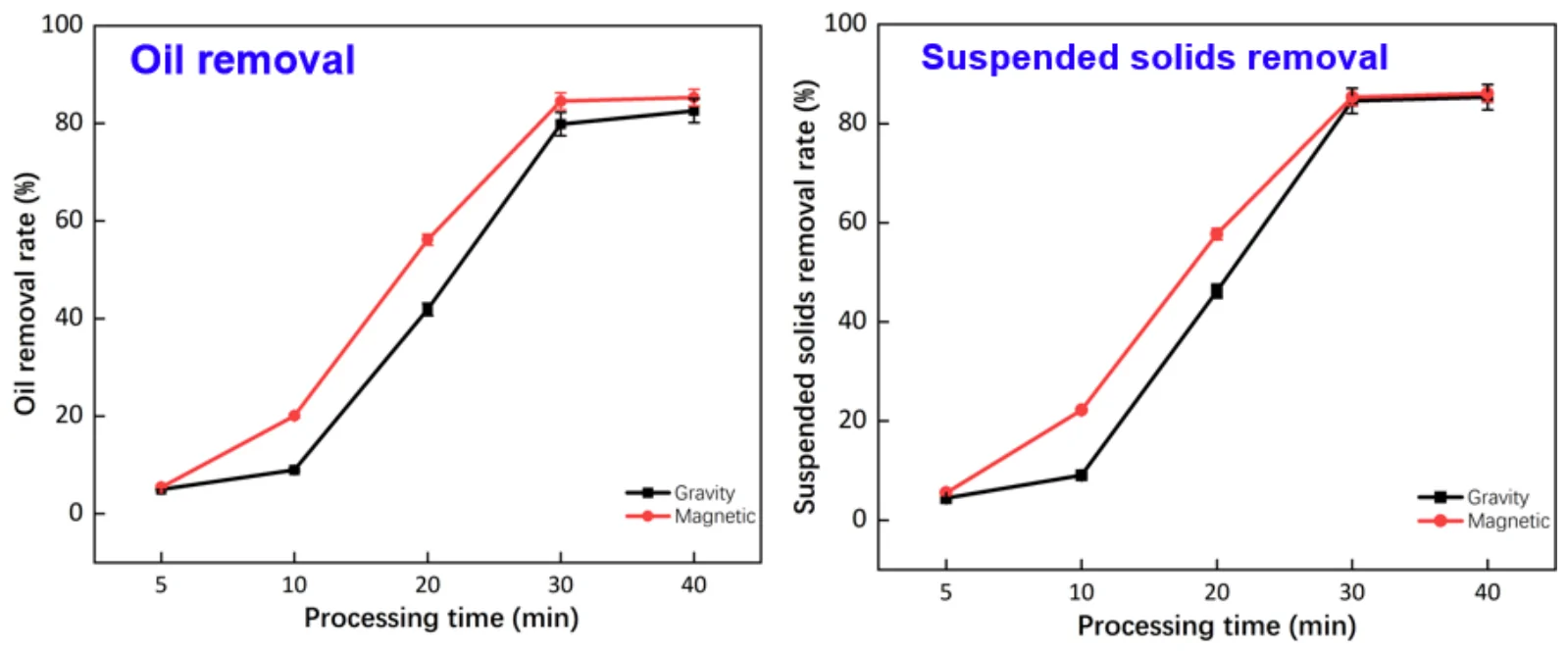
Time-dependent curve of efficiency of 3-FSNMN4-C for composite flooding wastewater. (**left**) Oil removal (**right**) Suspended soilds removal.

Through the FSNMN-c treatment of combined flooding wastewater experiment, it can be found that the treatment effect of the magnetic field experimental group is slightly stronger than that of the gravity field experimental group within 30 min. The additional external force of the magnetic field accelerates the migration of flocs, so experiments are carried out to verify the relationship between treatment time and treatment effect. The treatment agent is 3-FSNMN4-C, and the dosage is 20 mg/L. The treatment time groups without mutual interference are set respectively. The measured data are sampled according to the respective treatment time, and Figure 13 is obtained.

It can be seen from Figure 13 that when 3-FSNMN_4_-C was just added for 5 min, both groups did not complete demulsification and flocculation. After 10 min, the effect was improved, and the magnetic group was stronger than the gravity group, with a large gap. After 30 min, the increase in efficiency slowed down with time. After 30–40 min, the demulsification efficiency of the two groups was close. The demulsification efficiency of the magnetic group was higher than that of the gravity group, but the gap was narrowed.

Magnetic fields can significantly shorten sedimentation time, especially in high-viscosity wastewater systems where the advantage is even more pronounced. Furthermore, the magnetic force acting on the interior of flocs can promote further aggregation of oil droplets and solid particles, forming denser flocs and reducing their water content. To sum up, under the condition of magnetic field, FSNMN-C can treat ASP flooding wastewater faster, and its demulsification and flocculation effects are stronger than those of gravity natural settlement. It takes less time to achieve the same treatment efficiency, and it can achieve higher treatment efficiency in the same time.

## 4. Discussion

Mechanism analysis of ASP flooding wastewater treatment by FSNMN-C: the target objects of oilfield sewage treatment mainly include oil phase or oil drop and solid suspended solids. The main treatment mechanisms are the demulsification mechanism and flocculation mechanism.

### 4.1. Demulsification Mechanism

The composite flooding technology reduces the surface interfacial tension of the oil–water system to a very low value through the action of efficient surfactants. After absorbing the formation fluid, asphaltene and other natural surfactants enter the oil–water interface, enhancing the interfacial film and increasing the interfacial tension at the same time. When the oil well-produced liquid enters the separation station on the ground, the produced liquid is physically and chemically treated by the three-phase separator to recover the oil phase and generate sewage. The interfacial tension in the sewage further rises. There is no continuous oil phase in the ASP flooding sewage, and the remaining oil phase exists in the form of emulsified charged oil droplets. The oil–water interfacial tension of the composite sewage sample was 34.35 Mn/m. There are two main functions for the stable existence of oil droplets in ASP flooding wastewater:

(1) The strong oil–water interface, the tough and elastic interface formed by the interaction of natural surfactants (such as asphaltene) and oil displacement agents in the formation and oil layer hinders the collision and aggregation of emulsified tiny oil droplets and forms large oil droplets.

(2) At higher zeta potential, clay particles and oil droplets adsorb oil recovery agents or formation materials with strong negative charge, forming a strong electrostatic repulsion between particles, greatly reducing the collision probability between particles.

FSNMN-C aggregates emulsified tiny oil droplets into large oil droplets and an oil phase, which needs to overcome the above effects. FSNMN-C is not only a demulsifier, but also a flocculant with a hyperbranched long-chain structure. As long as the molecules can enter the oil–water interface, the stable interface membrane can also be destroyed through flocculation accumulation and magnetic migration to complete the demulsification effect.

Static interfacial tension can directly measure the demulsification performance of the demulsifier, and also indicate the change in interface. Taking ASP flooding wastewater as the treatment object, the effect of materials on the static interfacial tension of ASP flooding wastewater was compared and evaluated. Adjust the dosage of the treatment agent, set the experimental temperature at 25 °C, and, after mixing for 30 s, quickly transfer the sample to the static interfacial tension meter for measurement.

**Figure 14:**
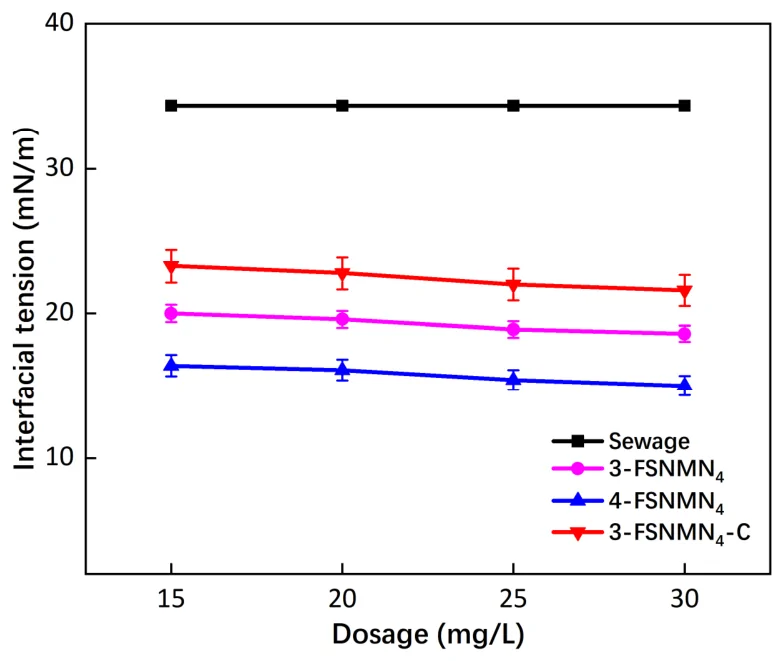
Changes in static interfacial tension of water after adding treatment agents.

Figure 14 shows that the 3-FSNMN_4_-C treatment agent with triethylenetetramine-branched third-generation magnetic hyperbranched molecules grafted with cationic long-chain can effectively reduce the interfacial tension, which further decreases with the amount of treatment agent, which is the result of the joint action of the rich ester group, primary amine group, secondary amine group, amide group and carbon chain contained in the magnetic hyperbranched long-chain structure of 3-FSNMN_4_-C. The ability of the same dosage of the treatment agent to reduce the interfacial tension reflects its demulsification ability. Table 3 and Table 4 can be obtained by combining the experimental data of the simulated emulsion performance. It can be found that the demulsification ability of the pure kerosene simulated emulsion 4-FSNMN_4_ is stronger than that of 3-FSNMN_4_ and 3-FSNMN_4_-C, which corresponds to the ability to reduce the interfacial tension. It also shows that with the increase in hyperbranched algebra, the intramolecular secondary amine and amide groups increase significantly and the ability to reduce interfacial tension and demulsification ability are improved; the content of hyperbranched molecular groups per unit mass of the FSNMN-C molecule is lower than that of the FSNMN molecule, the ability to reduce interfacial tension is lower than that of the FSNMN molecule, and the theoretical demulsification ability is weaker than that of the FSNMN molecule. The gravity group uses pure gravity settlement, whereas the magnetic group has an additional vertical magnetic field for sedimentation.

It can also be found in Table 3 and Table 4 that when treating a negatively charged kerosene emulsion, the demulsification ability of FSNMN-C is much stronger than that of FSNMN hyperbranched molecules with stronger ability to reduce interfacial tension. This is because 3-FSNMN_4_-C contains rich cationic groups, altering the behavior of hyperbranched parts at the oil–water interface and electrostatic adsorption to the surface of charged particles. When the dosage is 20 mg/L, the zeta potential can be reduced below −10 mV, which destroys the electrostatic repulsion balance between oil droplets, enhances the collision and coalescence of oil droplets, and greatly enhances the demulsification effect.

The effect of magnetic treatment goes further. The combined force of magnetic field and gravity field adds an external force to collide and coalesce more oil droplets. The magnetic force strengthens the migration of oil floc, improves the cleaning ability of residual oil droplets, and improves the demulsification ability. Summarizing the above analysis, it can be seen that the Demulsification Mechanism of FSNMN-C for composite flooding wastewater mainly has three parts.

(1) Destroy the oil–water interface of emulsified oil droplets in ASP flooding wastewater.

FSNMN-C is formed by multi-generation hyperbranched polyamide and cationic poly am-dac long chain. Hyperbranched groups, including some secondary amine, ester and amide groups, can reduce the oil–water interfacial tension. At the same time, the hyperbranched FSNMN-C adsorbs and penetrates into the oil–water interface film to produce demulsification.

(2) Reduce zeta potential of particles.

The long-chain part of cationic poly am-dac contains a large number of cationic groups. After FSNMN-c oil–water interface behavior and electrostatic adsorption, the zeta potential is reduced, the electrostatic repulsion is reduced, and the electrostatic stability between colloidal particles is destroyed. Demulsification occurs.

(3) The forced migration in the magnetic field, superimposed with the gravity field, enhances the collision and coalescence between particles, strengthens the cleaning ability of flocs, and enhances the demulsification effect.

### 4.2. Flocculation Mechanism

In the wastewater of ASP flooding in the oilfield, there are suspended solids, mainly composed of clay, rock cuttings and other formation minerals, which may also be solid substances added in the process of oil displacement. In the wastewater generated after passing through the three-phase separator, the suspended solids are stable in the form of charged colloidal particles. There are two main functions for the stable existence of suspended solids in ASP flooding wastewater.

(1) At high zeta potential, clay particles and oil droplets adsorb oil recovery agents or formation materials with strong negative charge, forming a strong electrostatic repulsion between particles. There are strong diffusion electric double layers outside various colloidal particles, with low collision probability between particles, not easy to gather.

(2) The polymer can enhance the suspension stability of viscosity. The residual anionic polymer in ASP flooding wastewater after oil–water separation still has a certain viscosity, and some solid particles adsorbed to ASP flooding wastewater form stable negative colloidal particles, which enhance electrostatic repulsion, hinder the collision between colloidal particles, reduce the intensity of colloidal particle movement, and balance the gravity sedimentation. Through measurement, the zeta potential of the sewage sample from the combined flooding is found to be −37 mV and the dynamic viscosity is 2.27 mPa·s.

The coagulation and sedimentation of colloidal particles in FSMN-C suspended solids require overcoming the aforementioned influences. FSNMN-C is not only a flocculant, but also a cationic demulsifier with a hyperbranched long-chain structure. The long chain is interlaced to form a flocculent structure after the interaction of long-chain adsorption gel and electrostatic adsorption. Destroy the stable suspension of suspended solids and colloidal particles, and separate them under the action of magnetic field and gravity field.

**Figure 15:**
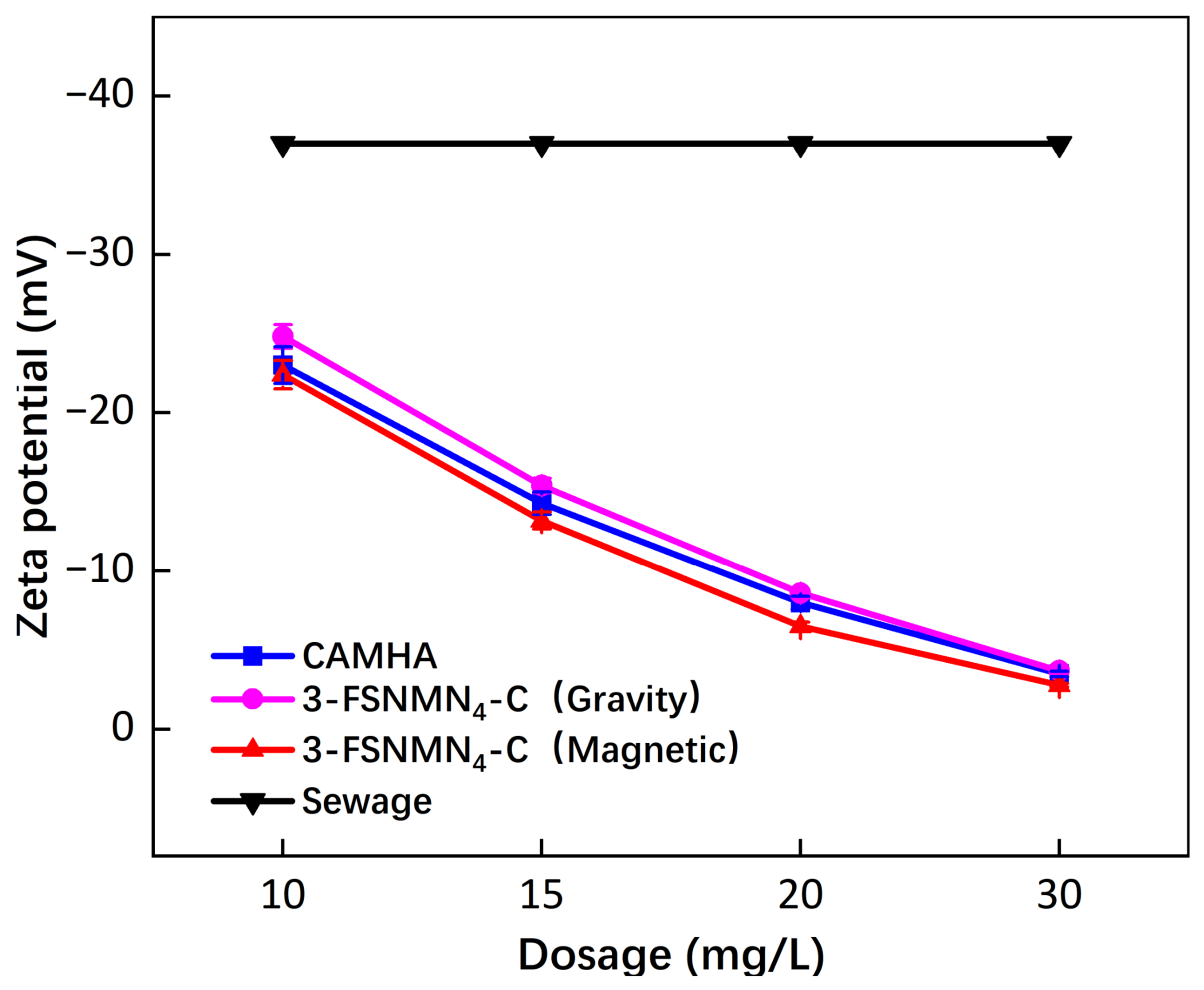
Zeta potential change after adding treatment agent to composite flooding wastewater.

The effects of CAMHA and 3-FSNMN_4_-C on the zeta potential of ASP flooding wastewater were evaluated. Adjust the dosage of the treatment agent, set the experimental temperature at 25 °C, add the treatment agent, mix well and stand for 30 min, take the supernatant to measure the data, and get Figure 15.

Figure 15 shows that the absolute value of zeta potential can be effectively reduced by the 3-FSNMN_4_-C treatment agent with triethylenetetramine-branched third-generation magnetic hyperbranched molecules grafted with cationic long chains. The absolute value of zeta potential further decreases with the dosage of treatment agent, which is due to the role of hydrolyzable cationic groups in the structure of the magnetic hyperbranched cationic polymer in 3-FSNMN_4_-C. The reduction in the absolute value of zeta potential by the same dosage of treatment agent reflects its ability to destroy the electrostatic balance and electrostatic flocculation. Table 5 and Table 6 are obtained by combining the experimental data of a simulated suspension performance.

It can be found that the treatment capacity of 3-FSNMN-C for the wastewater of a negative electric suspension system such as ASP flooding wastewater is stronger than that of the electrically neutral suspension system. The cationic long chain has its own polyacrylamide chain hydration adsorption and electrostatic adsorption to the surface of colloidal particles, which is inserted into its hydration double-layer, destroying the negative electric environment on the surface and reducing the absolute value of zeta potential. When it is reduced to −10 mV, electrostatic repulsion can no longer hinder the collision and aggregation of colloidal particles, leading to the formation of various network structures, such as “tail snakes” and “interlaced networks” formed by cationic long chains connecting each colloidal particle, which in turn form flocculated aggregates. Electrostatic flocculation and adsorption flocculation work together to build a bridge for net capture. The flocs change from less to more and from small to large to produce a net capture effect. Driven by the magnetic field and gravity field, more colloidal particles will be netted and settled.

However, when the dosage was 20 mg/L, the treatment effect was weaker than 15 mg/L. The dosage of macromolecular polymer treatment agent needs to be balanced with the side effects brought by viscosity, and the effect of the magnetic field reduces the effect of viscosity increase. The dynamic viscosity of ASP flooding-produced fluid can reach more than 3.0 MPa·s, and the ASP flooding sewage after oil–water separation also retains a high dynamic viscosity. The dynamic viscosity was measured immediately after the treatment agent was added into the ASP flooding sewage and mixed for 30 s, and Table 7 was obtained.

The average molecular weight of 3-FSNMN_4_-C is about 4.7 million. It can be seen in Table 7 that the viscosity of ASP flooding wastewater is similar to that of CAMHA, a cationic polymer with an average molecular weight of 4 million. With the increase in dosage, the dynamic viscosity first decreases and then increases, and the dynamic viscosity has a minimum value, which corresponds to the declining trend of 20 mg/L treatment performance in Table 7. This is because when FSNMN-C and CAMHA are added to the ASP flooding sewage, the viscosity produced by themselves is low when the dosage is low, and electrostatic adsorption and flocculation occur with the negatively charged hydrated colloidal particles, and because the net trapping function adsorbs the suspended solids, it also takes away the anionic/non-ionic polymer adsorbed on the surface of the suspended solids, which together reduces the content of anionic/non-ionic polymer in the sewage, thus reducing the viscosity. When the dosage of FSNMN-C and CAMHA exceeded the optimal value, the effect of increasing viscosity exceeded the effect of the reducing viscosity of adsorbed floc gel particles, and the overall viscosity increased.

The magnetic field sedimentation group can alleviate the negative effect of viscosity increase at high dosage, and the resultant force of flocculation sedimentation with a gravity field is stronger, the sedimentation is more thorough, and the flocculation effect is stronger. When the optimal dosage for the gravity field is exceeded, the magnetic field can obtain better flocculation and sedimentation effects. From the above analysis, it can be seen that the flocculation mechanism of FSNMN-C mainly has three parts.

(1) Reducing zeta potential of suspended colloidal particles in ASP flooding wastewater.

FSNMN-C hyperbranched cationic chain is adsorbed to the surface of suspended colloidal particles through adsorption and electrostatic adsorption, reducing the negative charge on the surface of colloidal particles, while destroying the stable hydration double electric layer. Multiple suspended colloidal particles collide and gather, forming flocs with long chains in a net. When settling, the net catching effect occurs, and the suspended colloidal particles are further flocculated.

(2) Reduce the polymer content and viscosity of ASP flooding wastewater at appropriate dosage.

FSNMN-C hyperbranched cationic straight chain polymer can directly initiate the flocculation of suspended colloidal particles formed by solid particles and anionic polymers through carbon chain adsorption and electrostatic attraction, reduce the dynamic viscosity of ASP flooding wastewater, directly reduce the suspension stability of wastewater, and promote flocculation and sedimentation.

(3) Under the action of an external magnetic field, it can directly enhance the occurrence of sedimentation, promote the sedimentation, strengthen the effect of suspended colloidal particle sedimentation and net-catching sedimentation, and improve the flocculation effect.

## 5. Conclusions

The magnetic hyperbranched molecule FSNMN was synthesized by iterative reaction method, which has good demulsification ability for electroneutral emulsion. Then cationic polymer CAMHA was synthesized, which has good flocculation ability for negative suspension and demulsification ability for negative emulsion. Finally, the synthesized cationic polymer CAMHA was grafted to the magnetic hyperbranched molecule FSNMN, which has a good treatment effect for negative emulsion and suspension. The demulsification and flocculation experiments of ASP flooding wastewater show that the magnetic hyperbranched polyamide cationic polymer has good demulsification and flocculation ability for the treatment of ASP flooding wastewater. The synthesis conditions of 3-FSNMN_4_-C were as follows: CAMHA was slowly added to the FSNMN dispersion, the final mass ratio was 1:1, the reaction time was 4 h, and the reaction temperature was 40 °C. The average molecular weight of 3-FSNMN_4_-C is 4.7 million, the cationic degree is 20.5%, and the saturation magnetization is 20 emu/g.

(1) 4-FSNMN_4_-C and 4-FSNMN_5_-C have the strongest ability to treat ASP flooding wastewater. When treated with a magnetic field for 30 min and a dosage of 20 mg/L, the oil removal rate can reach 90.27%, and the suspended solids removal rate can reach 91.32%. 3-FSNMN_4_-C, which is the optimal product for synthesis yield and treatment effect, its performance is slightly inferior. The oil removal rate can reach 84.52% and the suspended solids removal rate can reach 84.57% after magnetic field treatment for 30 min and with a dosage of 20 mg/L.

(2) The demulsification mechanism of magnetic hyperbranched cationic polymer FSNMN-C on composite flooding wastewater can destroy the oil–water interface, reduce the zeta potential and reduce the viscosity of wastewater. Under the action of magnetic field, oil droplets and colloidal particles are forced to accelerate coalescence and form flocs. Magnetic field treatment can enhance the processing efficiency of FSNMN-C molecules. Under the condition of magnetic field, FSNMN-C can treat ASP flooding wastewater faster. The demulsification and flocculation effects are stronger than those of gravity natural settlement, and higher treatment efficiency can be achieved in the same time frame.

(3) Currently, there are limitations such as the homogeneity of wastewater components, limited experimental modes, unknown cost-effectiveness, and insufficient long-term stability. Subsequent research will focus on conducting treatment experiments on ASP wastewater from different sources and with different properties, designing and building continuous-flow magnetic separation treatment devices, verifying the treatment effect and operational stability of the materials in actual industrial processes, optimizing the synthesis process (such as using cheap raw materials and simplifying reaction steps) to reduce material costs, conducting long-term environmental safety assessments of the materials, including structural changes after long-term cyclic use, heavy metal leaching risks, and ecological toxicity, exploring coupling processes between the materials and advanced oxidation, membrane separation, and other technologies, and further improving treatment efficiency and effluent quality.

## Figures and Tables

**Table 1:** Most suitable formation conditions (top 5 groups).

SN	Reaction Time/h	Reaction Temperature/°C	Reactant Ratio	Suspended Solids Removal Rate/%					
				N_1_	N_2_	N_3_	N_4_	N_5_	Satisfaction
1	4.1	40.5	1.0	84.5	90.5	92.9	94.8	94.5	1.0
2	4.0	45.8	1.1	84.7	90.8	93.1	94.8	94.5	1.0
3	3.4	33.5	1.8	85.4	90.5	93.2	94.9	94.5	1.0
4	4.0	38.1	1.2	84.6	90.5	93.0	94.8	94.6	1.0
5	3.1	39.0	1.8	86.1	91.3	93.6	95.1	94.5	1.0

**Table 2:** Cationic degree of FSNMN-C.

Material	Average Molecular Weight/Ten Thousand	Cationic Degree/%
CAMHA	413.0	21.0
3-FSNMN_1_-C	439.9	20.7
3-FSNMN_2_-C	439.9	21.4
3-FSNMN_3_-C	458.2	20.6
3-FSNMN_4_-C	469.7	20.5
3-FSNMN_5_-C	499.5	19.6

**Table 3:** Demulsification efficiency (dosage 20 mg/L).

Treating Agent	Condition	Zeta Potential/mV
None	gravity	−37.0
CAMHA	gravity	−8.0
3-FSNMN_4_-C	gravity	−8.6
3-FSNMN_4_-C	magnetic	−6.5

**Table 4:** Zeta potential after demulsification (dosage 20 mg/L).

Treating Agent	Condition	Oil Removal Rate/%	Oil Removal Rate/%
3-FSNMN_4_	gravity	43.2	53.7
4-FSNMN_4_	gravity	45.8	57.7
3-FSNMN_4_-C	gravity	88.6	54.9
3-FSNMN_4_-C	magnetic	93.8	64.9

**Table 5:** Comparison of flocculation efficiency with dosage of 15 mg/L.

Treating Agent	Condition	Negatively Charged Suspension/%	Electroneutral Suspension/%
CAMHA	gravity	91.60	80.70
3-FSNMN_4_-C	gravity	93.88	80.38
3-FSNMN_4_-C	magnetic	94.72	86.48

**Table 6:** Comparison of flocculation efficiency with dosage of 20 mg/L.

Treating Agent	Condition	Negatively Charged Suspension/%	Electroneutral Suspension/%
CAMHA	gravity	88.50	79.30
3-FSNMN_4_-C	gravity	87.72	80.13
3-FSNMN_4_-C	magnetic	92.35	86.24

**Table 7:** Dynamic viscosity of ASP flooding wastewater after adding treatment agent.

Treating Agent	Condition	10 mg/L^−1^	15 mg/L^−1^	20 mg/L^−1^
		Dynamic Viscosity/mPa·s		
None	gravity	2.27 mPa·s		
CAMHA	gravity	2.04	1.58	1.90
3-FSNMN_4_-C	gravity	1.96	1.50	1.74
3-FSNMN_4_-C	magnetic	1.92	1.50	1.72

## Data Availability

Data is contained within the article. The original contributions presented in this study are included in the article. Further inquiries can be directed to the corresponding author.

## Abbreviations

The following abbreviations are used in this manuscript:

FSNMN-C	Abbreviation of hyperbranched cationic polymer with nano ferric oxide core
FSNMN	Abbreviation of hyperbranched polymer with nano Fe_3_O_4_ core
CAMHA	Cationic polymer synthesized by acrylamide, acryloxyethyl trimethylammonium chloride and maleic anhydride
TEOS	Tetraethyl silicate
APTES	γ-Aminopropyltriethoxysilane
